# Exemptions and Limited Institutional Review Board Review: A Practical Look at the 2018 Common Rule Requirements for Exempt Research

**DOI:** 10.31486/toj.19.0095

**Published:** 2020

**Authors:** Amelia Walch-Patterson

**Affiliations:** Human Research Protection Program, Ochsner Clinic Foundation, New Orleans, LA

**Keywords:** *Clinical trials data monitoring committees*, *confidentiality*, *ethics committees–research*, *legislation*, *privacy*, *research subjects*

## Abstract

**Background:** The revised Common Rule sought to modernize an outdated regulatory framework, provide clarity to the research community about the application of regulations, and reduce regulatory burden. From the advance notice of proposed rulemaking in 2011 to the implementation of the Final Rule, a significant amount of commentary and opinion was generated about the rules that govern most federally funded human subjects research.

**Methods:** This article provides insight into the changes to the regulatory framework for low-risk research, clarifies when exemptions can be applied, and explains the use of limited institutional review board (IRB) review.

**Results:** In attempting to fulfill the objectives of reducing regulatory burden, freeing IRB administrative resources, and protecting human subjects, the new regulations acknowledge low-risk research and privacy concerns, as well as the increased use of biospecimens. In the Final Rule, the Office for Human Research Protections updated the definition of human subject and expanded the exemption framework. The definition of human subject in the Final Rule includes biospecimens, and the new exemption framework includes expanded definitions, modifications to existing exemption categories, the creation of new categories, and the creation of a new concept called limited IRB review. The expanded exemption framework was designed to help alleviate the regulatory burdens of low-risk research.

**Conclusion:** Whether the revised regulations will meet the needs of the research community and human subject participants is unknown. While the revised Common Rule includes some welcome modifications and additions, the changes have also introduced new concepts that are not fully elucidated and have therefore introduced new ambiguities.

## INTRODUCTION

In January 2017, the first major revisions to the Federal Policy for the Protection of Human Subjects—found at 45 CFR §46 and known as the Common Rule—were signed into law. The path to these new regulations began in 2011 when the US Department of Health and Human Services (HHS), the federal agency tasked to “enhance and protect the health and well-being of all Americans,”^1^ announced a proposal to improve human subjects protections. The advance notice of proposed rulemaking (ANPRM) noted that research, research methodologies, technology, and research volume had increased and evolved considerably over the years, and modernized regulations were needed to help facilitate these new research dynamics.^2^

Of particular consideration was the review process for minimal-risk studies. Federal regulations define minimal risk as “…the probability and magnitude of harm or discomfort anticipated in the research are not greater in and of themselves than those ordinarily encountered in daily life or during the performance of routine physical or psychological examinations or tests.”^3^

Minimal-risk studies can include surveys, interviews, and medical record reviews that are sufficiently low risk as to be deemed exempt from the regulations. However, minimal-risk research sometimes must undergo convened institutional review board (IRB) review,^4^ in part because reviewers have found the regulations “vague and difficult” to apply,^2^ leading to increased review times and variation among IRBs in their application of the regulations.^5-7^

If the research regulations were examined as tiers by risk and requirements, they could be summarized as follows:

1. Exempt–low risk, not requiring adherence to the regulations, including research informed consent, review and approval of changes to the research, or annual continuing review
2. Expedited–not greater than minimal risk, requiring consideration for research informed consent or waivers; IRB review and approval, including changes to the research; annual IRB review; and reporting of certain types of noncompliance
3. Full committee review–greater than minimal risk, requiring a convened committee, review and approval of changes to the research, sometimes full committee review of those changes, and annual continuing review

Although an IRB can strive to apply the least burdensome regulatory requirement, doing so can be a challenge.

The revised regulations needed to provide clarity to the research community, reduce the regulatory burden, and free IRB administrative resources while continuing to protect human subjects and their identifiable information and biospecimens. The revision process took several years. Updating the regulations for human subjects research took 7 years to develop and nearly a decade to be fully enacted.

## NOTICE OF PROPOSED RULEMAKING: INTRODUCTION OF EXCLUSIONS

After 4 years of soliciting comments from researchers and the public, HHS, in conjunction with other public agencies, announced the proposed new framework via a 2015 notice of proposed rulemaking (NPRM).^8^ To address the issue of regulatory burden for low-risk research, one of the proposals in the NPRM was the creation of a new class of research called *exclusions*. The proposal in the ANPRM was to call these research categories *excused*.^2^ The NPRM exclusions were designed to be completely excluded from the regulations and not require any type of review mechanism. The NPRM proposed these exclusions because the associated study types met one or more of the following criteria: they were of sufficiently low risk, were of extreme importance to the government, or would not impact subjects’ rights (Table 1). The exclusions were purported to provide clarity to activities sometimes construed as research.

**Table 1.** Proposed Exclusions in the 2015 Notice of Proposed Rulemaking^8^

Category	Exclusion	2018 Common Rule Adoption
Exclusion of activities that are deemed not research	Program improvement activities	Not meaningfully addressed
	Oral history, journalism, biography, and historical scholarship activities	Oral history, journalism, biography, literary criticism, legal research, and historical scholarship deemed not research^a^
	Criminal justice activities	Deemed not research^a^
	Quality assurance and quality improvement activities	Not meaningfully addressed
	Public health surveillance	Deemed not research^a^
	Intelligence surveillance activities	Deemed not research^a^
Exclusion of activities that are low risk and already subject to independent controls	Educational tests, survey procedures, interview procedures, or observation of public behaviors	Included in exemption categories 1, 2, and 3
	Research involving the collection or study of information that has been or will be collected	Partially addressed by exemption category 4
	Research conducted by a government agency using government-generated or government-collected data	Included in exemption category 5
	Certain activities covered by HIPAA	Included in exemption category 4
The following activities are excluded because they are considered to be low-risk human subjects research activities that do not meaningfully diminish subject autonomy.	The secondary research use of a nonidentified biospecimen that is designed only to generate information about an individual that already is known	Not meaningfully addressed

^a^See 45 CFR §46.102(l)(1)-(4).^3^

HIPAA, Health Insurance Portability and Accountability Act.

The first 6 exclusions were classified as not being research at all, and the NPRM contended that these 6 exclusions would sizably reduce the regulatory burden on researchers and free resources that had been previously used to determine if a proposed project met the definition of research.^8^ Professor Zachary Schrag, professor of history at George Mason University, pointed out on his blog that the social sciences community was especially keen for this much-needed relief in areas of oral history and journalistic activities.^9,10^

The next 4 exclusions attempted to reclassify some exempt research—such as medical record reviews and educational tests and surveys—because they were considered sufficiently low risk and had other control mechanisms in place to ensure protections. The NPRM defined low risk as not involving any physical risks and having other risks minimized by controls already in place. The NPRM cited control mechanisms such as the Privacy Act, the Health Information Portability and Accountability Act (HIPAA), and the Paperwork Reduction Act. Again, these exclusions as proposed were designed to reduce the regulatory burden and the use of IRB administrative resources so that the IRB could focus on the mandate of protecting human subjects.

The final proposed exclusion was to allow nonidentified biospecimens to be used for secondary research without IRB oversight. The NPRM argued that such biospecimens only provided information about a donor's condition that was already known, so their use would not meaningfully diminish the subject's autonomy and could therefore be excluded from regulatory oversight.^8^

When President Barack Obama signed the Final Rule into law on January 19, 2017, however, no exclusions were included in the rule. The exclusions proposed in the NPRM were either not addressed in the Final Rule or were incorporated into the exemptions.

## NOTICE OF PROPOSED RULEMAKING: EXEMPTION CHANGES

In the NPRM, proposed updates to the exemption framework included the minor administrative change of relocating the exemptions from 45 CFR §46.101 to 45 CFR §46.104. To address the “vague and difficult” complaints about the existing exemption categories, another part of the proposal required the Secretary of HHS to develop a tool for reviewers to use when determining if a study met the exemption criteria. However, such a tool would require an institutional record-keeping system depending on how it was implemented, and according to published commentary in the Final Rule,^3^ this proposal was controversial. The research community suggested that it could not support the proposed tool without the chance to review and validate it.

The exemptions proposed in the NPRM addressed benign interventions, issues of identifiability of data gathered during routine clinical procedures, and storage and maintenance of biospecimens for later research.^3^ One of the existing exemptions—the use of educational tests, survey or interview procedures, or observation of public behavior—was moved to the proposed exclusion categories in the NPRM. The exemptions were then recategorized in a similar way that exclusions were categorized:

1. Low-risk interventions for which there would have been no other requirement
2. Research activities that would have required the application of privacy safeguards
3. Secondary research involving biospecimens and identifiable private information that would have required application of privacy safeguards, broad consent, and limited IRB review.^8^

## THE FINAL RULE

While the changes to the Common Rule were signed into law in January 2017, they did not take effect until January 2019. Initially, the published rules were to take effect on January 20, 2018, 1 year after being signed into law, with one provision regarding cooperative IRB review (also known as the single IRB mandate) to take effect on January 20, 2020. Despite many years of commentary and dialog on the ANPRM and NPRM, the research community was not ready for the myriad changes in the Final Rule. A proposal to delay the effective date was issued, and the Final Rule was effective on January 21, 2019, with the Cooperative Research provision, also known as single IRB, enacted in January 2020. The initially planned implementation date of 2018 has led to the original Common Rule being referred to as the Pre-2018 Requirements and the revised Common Rule being referred to as the 2018 Requirements.

In the Final Rule, after receiving much commentary on the proposed exclusions, the Office for Human Research Protections (OHRP) abandoned the concept altogether and opted instead to update the definition of human subject and to expand the exemption framework. Commenters focused on exclusions as a source of confusion rather than clarity, pointing out that exclusions would further complicate decisions about when to apply the Common Rule, asking who would determine the application of an exclusion category, and asking how exclusions would be documented.^3^

The new exemption framework included expanded definitions, modifications to existing exemption categories, the creation of new categories, and the creation of a new concept called limited IRB review. The definition of human subject in the Final Rule included biospecimens, and the expanded exemption framework was designed to help alleviate the regulatory burdens of low-risk research.

## LIMITED INSTITUTIONAL REVIEW BOARD REVIEW

Limited IRB review requires that certain exempt research be reviewed by an IRB chair or designee for privacy and confidentiality under requirements in 45 CFR §46.111(a)(7) or §46.111(a)(8).^11^ The regulations at §46.111(a)(7) state, “When appropriate, there are adequate provisions to protect the privacy of subjects and to maintain the confidentiality of data,” and §46.111(a)(8) states, “… (iii) If there is a change made for research purposes in the way the identifiable private information or identifiable biospecimens are stored or maintained, there are adequate provisions to protect the privacy of subjects and to maintain the confidentiality of data.” This process is only applicable to certain new provisions in the exempt categories 2, 3, 7, and 8.

However, the Final Rule provides little detail about what constitutes protecting privacy and maintaining confidentiality under limited IRB review. A privacy and confidentiality review may cover the risks of deidentified information that can be reidentified; the extent to which the information will be shared or transferred to a third party or otherwise disclosed or released; the potential retention period of the information; security controls that are in place to protect the confidentiality and integrity of the information; and the potential risk of harm to individuals if the information is lost, stolen, or compromised.^3^ The preamble of the Final Rule states that the Secretary of HHS is committed to issuing guidance on limited IRB review.

## EXEMPTION CATEGORIES: PRE-2018 REQUIREMENTS VS 2018 REQUIREMENTS^12,13^

Each change in the exemption structure was meant to address the need for modernization, the lack of clarity for researchers and administrators, and public concern about the use of identifiable data and biospecimens. The following sections discuss the changes in each exemption category and the use of limited IRB review.

### Exemption Category 1 – Educational Practices

In keeping with the objective of reducing regulatory burden and providing clarity, the regulators left exemption category 1 largely unchanged (Table 2) and added no provision for privacy safeguards. The only added burden for the reviewer is to determine if an educational research protocol would be disruptive to a student's learning environment. However, commentary indicated that *disruptive* might be difficult to predict.^3^ Until the Final Rule has been applied in numerous settings and analysis is done on the impact of this change, the research community will not understand if the new wording has reduced or introduced confusion.

**Table 2.** Exemption Category 1 – Educational Practices

Pre-2018 Requirements^12^ (prior exemption category at 45 CFR §46.101)	Final Rule^13^ (new exemption category at 45 CFR §46.104)
(1) Research conducted in established or commonly accepted educational settings involving normal educational practices, such as (i) research on regular and special education instructional strategies, or (ii) research on the effectiveness of or the comparison among instructional techniques, curricula, or classroom management methods.	(1) Research conducted in established or commonly accepted educational settings that specifically involves normal educational practices that are not likely to adversely impact students' opportunity to learn required educational content or the assessment of educators who provide instruction. This includes most research on regular and special education instructional strategies, and research on the effectiveness of or the comparison among instructional techniques, curricula, or classroom management methods.

### Exemption Category 2 – Surveys, Interviews, Educational Tests, and Public Observations

The ANPRM envisioned developing this exemption into an excused category that dealt only with competent adults and had privacy safeguards built in.^2^ The NPRM classified this exemption as an exclusion in the category “Exclusion of activities that are low risk and already subject to independent controls (Table 1).” In the Pre-2018 Requirements, the types of research covered by this exemption could not be considered exempt unless the data were essentially anonymous or the researcher set up a method to indefinitely maintain confidentiality and the participants’ responses did not pose substantive nonphysical risks. In the Final Rule, the wording about identifiability was clarified, threats to educational advancement were included as an unacceptable harm, and researchers were given leeway to include identifiable information if sufficient precautions are taken (Table 3). By applying limited IRB review, a research protocol that includes identifiers could qualify for exempt status if it included privacy and confidentiality protection measures. The preamble of the Final Rule states that “if the information collected is both identifiable and sensitive or potentially harmful, the safeguards offered by the limited IRB review requirements at §__.111(a)(7) apply.” As it reads, the regulation does not require that the information collected under the exemption category be sensitive or potentially harmful, only that limited IRB review is applicable if subject identity is readily ascertainable. Because no guidance has been issued, an IRB reviewer could reasonably make one of three decisions:

1. Determine exemption and apply the 45 CFR §46.111(a)(7) criteria if the protocol includes sufficient information that confidentiality and privacy will be maintained.
2. Apply expedited criteria under 45 CFR §46.110 and issue a consent waiver under §46.116(e) if the protocol does not include adequate information about how privacy and confidentiality will be maintained.
3. Ask the researcher to provide sufficient information to make the exempt determination and apply 45 CFR §46.111(a)(7) criteria.

**Table 3.** Exemption Category 2 – Surveys, Interviews, Educational Tests, and Public Observations

Pre-2018 Requirements^12^ (prior exemption category at 45 CFR §46.101)	Final Rule^13^ (new exemption category at 45 CFR §46.104)
(2) Research involving the use of educational tests (cognitive, diagnostic, aptitude, achievement), survey procedures, interview procedures or observation of public behavior, unless (i) information obtained is recorded in such a manner that human subjects can be identified, directly or through identifiers linked to the subjects; and (ii) any disclosure of the human subjects’ responses outside the research could reasonably place the subjects at risk of criminal or civil liability or be damaging to the subjects’ financial standing, employability, or reputation.	(2) Research that only includes interactions involving educational tests (cognitive, diagnostic, aptitude, achievement), survey procedures, interview procedures, or observation of public behavior (including visual or auditory recording) if at least one of the following criteria is met: (i) the information obtained is recorded by the investigator in such a manner that the identity of the human subjects cannot readily be ascertained, directly or through identifiers linked to the subjects; (ii) any disclosure of the human subjects' responses outside the research would not reasonably place the subjects at risk of criminal or civil liability or be damaging to the subjects' financial standing, employability, educational advancement, or reputation; or (iii) the information obtained is recorded by the investigator in such a manner that the identity of the human subjects can readily be ascertained, directly or through identifiers linked to the subjects, and an IRB conducts a limited IRB review to make the determination required by §46.111(a)(7).

IRB, institutional review board.

These options do not offer the relief regulators promised in the NPRM. IRB reviewers still face uncertainty about how to apply the regulations.

### Exemption Category 3 – Benign Behavioral Interventions

This exemption category underwent substantial modification and expansion (Table 4). Under the Pre-2018 Requirements, this category was a variation of category 2, but the Final Rule overhauled this category and introduced the concept of benign behavioral intervention. A benign behavioral intervention is described in the exemption as being brief in duration, harmless, painless, not physically invasive, and not likely to have a significant adverse lasting impact on the subjects; further, the investigator must have no reason to think the subjects will find the interventions offensive or embarrassing. This exemption was designed to allow for low-risk research interventions and data collection in adults. In the Final Rule preamble, an example application of this new category is comparing students who take a test while listening to music to students who take the test in silence.^3^

**Table 4.** Exemption Category 3 – Benign Behavioral Interventions

Pre-2018 Requirements^12^ (prior exemption category at 45 CFR §46.101)	Final Rule^13^ (new exemption category at 45 CFR §46.104)
(3) Research involving the use of educational tests (cognitive, diagnostic, aptitude, achievement), survey procedures, interview procedures, or observation of public behavior that is not exempt under paragraph (b)(2) of this section, if: (i) the human subjects are elected or appointed public officials or candidates for public office; or (ii) federal statute(s) require(s) without exception that the confidentiality of the personally identifiable information will be maintained throughout the research and thereafter.	(3)(i) Research involving benign behavioral interventions in conjunction with the collection of information from an adult subject through verbal or written responses (including data entry) or audiovisual recording if the subject prospectively agrees to the intervention and information collection and at least one of the following criteria is met: (A) the information obtained is recorded by the investigator in such a manner that the identity of the human subjects cannot readily be ascertained, directly or through identifiers linked to the subjects; (B) any disclosure of the human subjects' responses outside the research would not reasonably place the subjects at risk of criminal or civil liability or be damaging to the subjects' financial standing, employability, educational advancement, or reputation; or (C) the information obtained is recorded by the investigator in such a manner that the identity of the human subjects can readily be ascertained, directly or through identifiers linked to the subjects, and an IRB conducts a limited IRB review to make the determination required by §46.111(a)(7).
	(iii) If the research involves deceiving the subjects regarding the nature or purposes of the research, this exemption is not applicable unless the subject authorizes the deception through a prospective agreement to participate in research in circumstances in which the subject is informed that he or she will be unaware of or misled regarding the nature or purposes of the research.

IRB, institutional review board.

Limited IRB review is required if the benign behavioral intervention research will collect identifiable information, so the IRB reviewer is again put in the position of having to determine if the protocol meets the qualifications for 45 CFR §46.111(a)(7) privacy and confidentiality requirements. If the reviewer cannot easily make this determination, either the review time (by asking the investigator to amend the protocol to address the issue) or the regulatory burden (by applying expedited criteria and consent waivers) will increase.

### Exemption Category 4 – Secondary Research Uses of Identifiable Private Information or Identifiable Biospecimens

The Final Rule permits data or biospecimens that have been or will initially be collected in the course of routine medical care to be used for research under this exemption (Table 5). Because HIPAA covers the data and biospecimens, limited IRB review is not necessary. This change was welcomed because it exempts low-risk research that is covered by other protection mechanisms. Previously, institutions were reluctant to apply this exemption category because it did not have a HIPAA provision; institutions instead opted to apply expedited category 5.

**Table 5.** Exemption Category 4 – Secondary Research Uses of Identifiable Private Information or Identifiable Biospecimens

Pre-2018 Requirements^12^ (prior exemption category at 45 CFR §46.101)	Final Rule^13^ (new exemption category at 45 CFR §46.104)
(4) Research, involving the collection or study of existing data, documents, records, pathological specimens, or diagnostic specimens, if these sources are publicly available or if the information is recorded by the investigator in such a manner that subjects cannot be identified, directly or through identifiers linked to the subjects.	(4) Secondary research for which consent is not required: secondary research uses of identifiable private information or identifiable biospecimens, if at least one of the following criteria is met: (i) the identifiable private information or identifiable biospecimens are publicly available; (ii) information, which may include information about biospecimens, is recorded by the investigator in such a manner that the identity of the human subjects cannot readily be ascertained directly or through identifiers linked to the subjects, the investigator does not contact the subjects, and the investigator will not reidentify subjects; (iii) the research involves only information collection and analysis involving the investigator's use of identifiable health information when that use is regulated under 45 CFR §160 and 45 CFR §164, subparts A and E, for the purposes of “health care operations” or “research” as those terms are defined at 45 CFR §164.501 or for “public health activities and purposes” as described under 45 CFR §164.512(b); or (iv) the research is conducted by, or on behalf of, a federal department or agency using government-generated or government-collected information obtained for non-research activities, if the research generates identifiable private information that is or will be maintained on information technology that is subject to and in compliance with… the E-Government Act of 2002, if all of the identifiable private information…will be maintained in systems of records subject to the Privacy Act of 1974, and, if applicable, the information used in the research was collected subject to the Paperwork Reduction Act of 1995*.*

Note: Some sections of the 2018 regulation in the Final Rule are abbreviated in this table.

Another modernizing aspect of this exemption category is the provision that researchers agree not to contact or reidentify subjects. This provision addresses technologic advances that could easily reveal subjects’ identities even in anonymous data, thus compromising their privacy, but restricts an investigator from using any newly found identifiers.

Because of the changes to this category, many medical record reviews can be deemed exempt, thereby reducing regulatory burdens on researchers.

### Exemption Category 5 – Federal Research/Demonstration Projects

This exemption was updated and expanded to clarify federally conducted activities that are low risk (Table 6). In the words of the Final Rule preamble, “The goal of this proposed requirement is to promote transparency of federally conducted or supported activities affecting the public that are not subject to oversight under the Common Rule.”

**Table 6.** Exemption Category 5 – Federal Research/Demonstration Projects

Pre-2018 Requirements^12^ (prior exemption category at 45 CFR §46.101)	Final Rule^13^ (new exemption category at 45 CFR §46.104)
(5) Research and demonstration projects which are conducted by or subject to the approval of department or agency heads and which are designed to study, evaluate, or otherwise examine (i) public benefit or service programs; (ii) procedures for obtaining benefits or services under those programs; (iii) possible changes in or alternatives to those programs or procedures; or (iv) possible changes in methods or levels of payment for benefits or services under those programs.	(5) Research and demonstration projects that are conducted or supported by a federal department or agency, or otherwise subject to the approval of department or agency heads (or the approval of the heads of bureaus or other subordinate agencies that have been delegated authority to conduct the research and demonstration projects), and that are designed to study, evaluate, improve, or otherwise examine public benefit or service programs, including procedures for obtaining benefits or services under those programs, possible changes in or alternatives to those programs or procedures, or possible changes in methods or levels of payment for benefits or services under those programs. Such projects include, but are not limited to, internal studies by federal employees and studies under contracts or consulting arrangements, cooperative agreements, or grants.
	(i) Each federal department or agency conducting or supporting the research and demonstration projects must establish, on a publicly accessible federal website or in such other manner as the department or agency head may determine, a list of the research and demonstration projects that the federal department or agency conducts or supports under this provision. The research or demonstration project must be published on this list prior to commencing the research involving human subjects.

Note: Some sections of the 2018 regulation in the Final Rule are abbreviated in this table.

### Exemption Category 6 – Taste and Food Evaluation Studies

This exemption category dealing primarily with food taste and quality evaluations remained essentially unchanged (Table 7).

**Table 7.** Exemption Category 6 – Taste and Food Evaluation Studies

Pre-2018 Requirements^12^ (prior exemption category at 45 CFR §46.101)	Final Rule^13^ (new exemption category at 45 CFR §46.104)
(6) Taste and food quality evaluation and consumer acceptance studies, (i) if wholesome foods without additives are consumed or (ii) if a food is consumed that contains a food ingredient at or below the level and for a use found to be safe, or agricultural chemical or environmental contaminant at or below the level found to be safe, by the Food and Drug Administration or approved by the Environmental Protection Agency or the Food Safety and Inspection Service of the US Department of Agriculture.	(6) Taste and food quality evaluation and consumer acceptance studies, (i) if wholesome foods without additives are consumed, or (ii) if a food is consumed that contains a food ingredient at or below the level and for a use found to be safe, or agricultural chemical or environmental contaminant at or below the level found to be safe, by the Food and Drug Administration or approved by the Environmental Protection Agency or the Food Safety and Inspection Service of the US Department of Agriculture.

### Exemption Categories 7 and 8 – Storage and Maintenance of Identifiable Information or Identifiable Biospecimens/Secondary Research Requiring Broad Consent

Two new exemption categories envisioned in the NPRM were introduced in the Final Rule (Table 8). These categories allow an exemption for the storage, maintenance, and secondary research uses of identifiable data and biospecimens if certain provisions are applied, namely limited IRB review [45 CFR §46.111(a)(7)] and broad consent [45 CFR §46.111(a)(8)]. In the Pre-2018 Requirements, identifiable data or biospecimens collected during primary research required full committee review to be used in secondary research because no exemption or expedited review category for these data or biospecimens existed.

**Table 8.** New Exemption Categories 7 and 8 – Storage or Maintenance of Identifiable Information or Identifiable Biospecimens / Secondary Research Requiring Broad Consent

Final Rule^13^ (new exemption categories at 45 CFR §46.104)	
(7) Storage or maintenance for secondary research for which broad consent is required: storage or maintenance of identifiable private information or identifiable biospecimens for potential secondary research use if an IRB conducts a limited IRB review and makes the determinations required by 45 CFR §46.111(a)(8).	
(8) Secondary research for which broad consent is required: research involving the use of identifiable private information or identifiable biospecimens for secondary research use, if the following criteria are met: (i) broad consent for the storage, maintenance, and secondary research use of the identifiable private information or identifiable biospecimens was obtained in accordance with 45 CFR §46.116(a)(1) through (4), (a)(6), and (d); and (ii) documentation of informed consent or waiver of documentation of consent was obtained in accordance with 45 CFR §46.117; (iii) an IRB conducts a limited IRB review and makes the determination required by 45 CFR §46.111(a)(7) and makes the determination that the research to be conducted is within the scope of the broad consent referenced in paragraph (d)(8)(i) of this section; and (iv) the investigator does not include returning individual research results to subjects as part of the study plan. This provision does not prevent an investigator from abiding by any legal requirements to return individual research results.	

IRB, institutional review board.

These proposed exemptions drew criticism from the public and controversy in the research community.^3^ Researchers criticized the requirement of having to track subjects who decline consent for the collection of their information and/or specimens or decline consent for the potential research uses identified in the broad consent form. Anecdotal evidence suggests that many institutions also interpreted these requirements to be overly burdensome because of the financial investment for software tracking systems and training personnel that would be required to follow the rule.

Revised exemption category 4 offers a pathway for secondary research uses of these same biospecimens/data with limited IRB review if researchers wish to maintain the identifiability under a confidentiality and privacy plan. Alternatively, the researcher could use an honest broker to have the data/biospecimens deidentified and qualify for the category 4 exemption. Also, the revised Common Rule still offers the possibility of continuing to use full committee review with a waiver of consent. Both of these routes for secondary uses superficially appear less onerous than the requirements under categories 7 and/or 8, which would require more time and resources to develop the broad consent, consent subjects, and track consent preferences and data/biospecimens. Because of these requirements, whether exemption categories 7 and 8 and broad consent will ever be feasible is unclear.

## LOOKING FORWARD

According to the Belmont Report,^14^ a cornerstone document of human subjects research protection in the United States, three ethical principles must be considered in the evaluation of any research project: beneficence, respect for persons, and justice. Those principles are reflected in the changes to the exemptions and the addition of limited IRB review, but the problem of vagueness in the regulatory structure persists. Anyone—researcher, regulator, or reviewer—can make an argument for how these ethical principles are applied to their decisions.

The journey to modernize the regulations produced a great deal of commentary and opinion, and the Final Rule is now being operationalized by institutions that are trying to fulfill the same ethical principles based on the same regulations. Whether the Final Rule will achieve the goals of reducing regulatory burden and clarifying the regulations for low-risk research and privacy and confidentiality safeguards will take time to determine.

While exclusions were not adopted in the Final Rule, they might have provided some of the clarity researchers and IRB reviewers sought. On the other hand, adding a fourth tier to the exempt, expedited, full committee scheme may have introduced more confusion and IRB overreach, although this possibility was raised in commentary from the community^8^ and was not based on implementation at any institution.

Because exemptions have the benefit of not having to adhere to all the requirements of 45 CFR §46, they should be clear and unambiguous for researchers to qualify for and reviewers to determine. That situation has historically not been the case, and given the commentary from the NPRM, the lack of guidance from the OHRP, and the preamble of the Final Rule, complete clarity is unlikely to be achieved.

However, some victories were realized. Allowing for the caveat that OHRP guidance may state differently, limited IRB review appears to permit more research to qualify as exempt, thereby reducing regulatory burden. At medical institutions where researchers conduct a significant number of medical record reviews in which only clinical data are collected, those studies can now be deemed exempt with reliance on HIPAA as the privacy safeguard. IRB reviewers who previously applied expedited categories for surveys, tests, and interviews because identifiers needed to be collected can apply limited IRB review at 45 CFR §46.111(a)(7) without having to consider the rest of the regulations.

## CONCLUSION

Research, researchers, and research institutions are not homogenous. Standardization of how each of these entities approaches human subjects protection in research is difficult. Whether the revised regulations will meet the needs of the research community and human subject participants is unknown. While the revised Common Rule includes some welcome modifications and additions, the changes have also introduced new concepts that are not fully elucidated and have therefore introduced new ambiguities. Perhaps it would be worth exploring whether standardization via guidance and OHRP publications is unnecessary and let institutions and researchers decide for themselves how best to apply these new regulations.

## Notes

This article meets the Accreditation Council for Graduate Medical Education and the American Board of Medical Specialties Maintenance of Certification competencies for Patient Care, Medical Knowledge, and Systems-Based Practice.
